# Anti-Müllerian Hormone Gene Polymorphism is Associated with Clinical Pregnancy of Fresh IVF Cycles

**DOI:** 10.3390/ijerph16050841

**Published:** 2019-03-08

**Authors:** Cheng-Hsuan Wu, Shun-Fa Yang, Hui-Mei Tsao, Yu-Jun Chang, Tsung-Hsien Lee, Maw-Sheng Lee

**Affiliations:** 1 Institute of Medicine, Chung Shan Medical University, Taichung 40201, Taiwan; 97528@cch.org.tw (C.-H.W.); ysf@csmu.edu.tw (S.-F.Y.); 2 Women’s Health Research Laboratory, Changhua Christian Hospital, Changhua 50006, Taiwan; 3 School of Medicine, Kaohsiung Medical University, Kaohsiung 80708, Taiwan; 4 Department of Medical Research, Chung Shan Medical University Hospital, Taichung 40201, Taiwan; 5 Division of Infertility Clinic, Lee Womens’ Hospital, Taichung 406, Taiwan; cshn076@csh.org.tw; 6 Epidemiology and Biostatistics Center, Changhua Christian Hospital, Changhua 50006, Taiwan; 83686@cch.org.tw; 7 Department of Obstetrics and Gynecology, Chung Shan Medical University Hospital, Taichung 40201, Taiwan

**Keywords:** Anti-Müllerian hormone, Anti-Müllerian hormone type II receptor, AMH polymorphism, AMHRII polymorphism, IVF outcomes

## Abstract

The aim of this study was to examine the effects of single-nucleotide polymorphisms (SNPs) in the anti-Müllerian hormone (AMH) and AMH type II receptor (AMHRII) genes on in vitro fertilization (IVF) outcomes. In this prospective cohort study, we genotyped the AMH 146 T > G, AMHRII −482 A > G and AMHRII IVS1 +149 T > A variants in 635 women undergoing their first cycle of controlled ovarian stimulation for IVF. DNA was extracted from the peripheral blood of all participants, and the SNPs were genotyped by real-time polymerase chain reaction. The distributions, frequencies of genes, and correlation with clinical pregnancy of IVF were analyzed. The AMH 146 T > G G/G genotype in women was associated with a lower clinical pregnancy rate (T/T: 55.0%, T/G: 51.8%, G/G: 40.0%; *p* < 0.05). Women with the AMH 146 T > G GG genotype were half as likely to have a clinical pregnancy compared with women with TT genotypes (OR = 0.55, 95% CI: 0.34–0.88, *p* = 0.014). With multivariate analysis, the AMH 146 T > G GG genotype remains as a significant independent factor to predict clinical pregnancy (*p* = 0.014). No significant difference was found between AMHRII polymorphisms and clinical pregnancy outcomes of IVF. In conclusion, our results show that AMH 146 T > G seems to be a susceptibility biomarker capable of predicting IVF pregnancy outcomes. Further studies should focus on the mechanism of these associations and the inclusion of other ethnic populations to confirm the findings of this study.

## 1. Introduction

Anti-Müllerian hormone (AMH), also called Müllerian-inhibiting substance, is a member of the transforming growth factor-beta (TGF-β) superfamily [1]. In women, AMH is exclusively produced in the ovary by granulosa cells surrounding preantral and small antral follicles [2]. AMH inhibits the primordial to primary follicle transition and decreases follicle-stimulating hormone (FSH) sensitivity [3]. Studies in AMH knockout mice have shown that in the absence of AMH, primordial follicle recruitment increases and follicles exhibit an increased sensitivity to FSH [4]. Therefore, serum AMH levels are thought to reflect the size of the growing cohort of small follicles, which in turn reflects the ovarian reserve [5]. A systematic review has reported that AMH is a good indicator of ovarian response in women undergoing controlled ovarian hyperstimulation (COH) for in vitro fertilization (IVF) [6]. Indeed, AMH was strongly associated with ovarian response and oocyte yield [7,8], which is a known major determinant of pregnancy outcome and live births in IVF cycles [9]. 

The molecular function or signaling transduction of AMH depends on the AMH receptors on the AMH-responding cells. There are two kinds of AMH receptors; the AMH type II receptor (AMHRII) is highly specific, while the identity of the AMH type I receptor remains unclear [10]. AMHRII is expressed both in granulosa cells of follicles and on the ovarian surface epithelium [11], showing its primary role in paracrine or autocrine function of granulosa cells and might closely related to gonadotropin-regulated follicular growth. Our previous study suggested that low serum AMH levels might be associated with high follicular FSH levels and poor pregnancy outcome in IVF cycles [12]. Although AMH/AMRII system is mainly expressed in ovarian granulosa cells, the modulation effect of AMH/AMRII on gonadotropin-releasing hormone/FSH/estradiol may be correlated to the pregnancy outcome in IVF cycles. 

The role of serum AMH as a good marker of ovarian response after ovarian stimulation has been well established [13]; however, its value in predicting the likelihood of pregnancy of IVF has been controversial. Pacheco et al. showed that even in the very low AMH level group, the probability of getting pregnant was reasonable, especially if the patient’s age was not very advanced [14]. Two systematic reviews showed that AMH has weak associations with both clinical pregnancy and live birth rates in IVF cycles [15,16]. Taken together, AMH appears to be a weak independent predictor of pregnancy and live birth rates of IVF [17]. This suggests that factors such as female age and cycle length, other than the ovarian reserve, likely affect chances of getting pregnant [18]. Because of the importance of AMH in regulating FSH sensitivity in the ovary and follicular recruitment [19], we speculated that genetic variants in the AMH signaling pathway could be associated with pregnancy outcome in women undergoing IVF treatment. 

Kevenaar et al. surveyed the genetic variation in AMH/AMHRII and showed that the AMH 146 T > G and the AMHR II −482 A > G SNPs are associated with an increase in follicular phase estradiol levels in normoovulatory women [20]. However, no differences in the levels of the pituitary gonadotrophin hormones, luteinizing hormone (LH), and FSH, were observed among the AMH and AMHRII genotypes, suggesting a direct effect of AMH on the ovary [20]. In other words, the study suggested that both polymorphisms contribute to the individual variation in the FSH “threshold” and modulate intraovarian FSH sensitivity [20]. Another prospective observational study showed that among the G/G genotypes of the AMH 146 T > G polymorphism, basal FSH levels were higher in those with more than two previous IVF attempts [21]. In addition, these two polymorphisms have been associated with unexplained infertility [22], pathogenesis of polycystic ovary syndrome [23,24], and effects during COH in ART treatment [21,25,26,27,28]. Hence, genetic variants in both the AMH and AMHRII genes may affect hormone function during folliculogenesis and may affect the outcome of IVF treatment.

Six previous studies have reported the association between these polymorphisms and their effects during ovarian stimulation in IVF treatment. A study by Hanevik et al. showed no statistically significant association between the AMH and AMHRII polymorphisms and response to ovarian stimulation in 191 IVF cycles in a population in Norway [25]. Another study in Greece consisting of 151 subjects indicated that women with a wild type for the AMHRII polymorphism and more than two previous IVF attempts had a higher number of follicles [21]. The third study by Peluso et al. demonstrated that both the AMH and AMHRII polymorphisms were associated with the number of embryos produced, but no association was found with pregnancy rates in 186 infertile women in Brazil [26]. The fourth study of 300 women by Lazaros et al. showed that the AMHRII −482 A **>** G genetic variants were associated with the ovarian response to standard gonadotropin stimulation; however, no association was observed with clinical pregnancy rates of IVF in Greece [27]. Cerra et al. reported that no significant associations were found between the variants AMH 146 T > G (rs10407022) and AMHRII −482 A > G (rs2002555) with ovarian response regarding oocytes retrieved and live births in 603 IVF cycles in UK [28]. Yoshida et al., in 2014, reported that AMHRII −482 A > G SNP may be involved in the malfunction of follicular development during IVF treatment in Japanese women [29].

The different findings of the above studies urged us to investigate whether the AMH and AMHRII genetic variants influence the ovarian response and outcomes of women undergoing IVF in the Taiwan Han population. This genetic study will help to illustrate whether the AMH and AMHRII polymorphisms can be utilized as genetic markers to add value in the prediction of both ovarian response and IVF outcomes.

## 2. Materials and Methods 

### 2.1. Study Design and Subjects

The patient cohort in this prospective study was composed of 635 women undergoing their first IVF treatment cycle from January 2014 to December 2015. Eligibility inclusion criteria were: (1) women age ≤ 40 years old (range 30–38 years); (2) no previous ovarian surgery or pelvic radiation therapy; (3) undergoing the first IVF or intracytoplasmic sperm injection (ICSI) cycle; and (4) blastocyst transfer in the fresh cycle. A venous blood sample was drawn for DNA extraction with subsequent genotyping. Ethics approval (CS13194) was obtained from the Institutional Review Board of Chung Shan Medical University Hospital. All participants provided written informed consent. Clinical trial register number: ISRCTN12768989. Only Han Chinese people were recruited for this analysis.

We studied the effects of the following three single-nucleotide polymorphisms (SNPs) on IVF outcomes: AMH 146 T > G (rs10407022), AMHRII −482 A > G (rs2002555), and AMHRII IVS1 + 149 T > A (rs2272002). The SNPs were chosen based on previous work by Kevenaar et al. [20], the international HapMap project (http://hapmap.ncbi.nlm.nih.gov), and searches in the dbSNP (http://www.ncbi.nlm.nih.gov/snp). The relevant IVF outcome was clinical pregnancy, which was defined as the presence of an intrauterine gestational sac.

### 2.2. IVF Treatment Protocol

All patients who participated in the current study underwent the same long GnRH agonist stimulation protocol to avoid any bias in the association between the AMH and AMHRII polymorphisms and IVF outcomes. The details of the stimulation cycle procedure have been previously described [30]. The long protocol began with daily subcutaneous injections of 0.5 mg of leuprolide acetate (Lupron; Takeda Pharmaceutics, Konstantz, Germany) from cycle day 21 of the previous cycle. On cycle day 3, recombinant FSH (Gonal-F, Merck-Serono, Darmstadt, Germany) or highly purified FSH (Menopur; Ferring Pharmaceuticals) was administered via an individual set with flexible doses. Final oocyte maturation was triggered with 10,000 IU human chorionic gonadotropin (Profasi, Serono, Norwell, MA, USA) and oocyte retrieval was performed 36 to 38 h later. Fertilization was carried out either by conventional insemination or ICSI depending on the semen parameters. Fresh blastocyst transfer was performed throughout the study period.

### 2.3. DNA Extraction and Determination of Genotypes

Genomic DNA was extracted from EDTA anti-coagulated venous blood using a QIAamp DNA blood mini kit (Qiagen, Valencia, CA, USA), according to the manufacturer’s instructions described in detail previously [31]. DNA was dissolved in Tris-EDTA (TE) buffer (10 mM Tris and 1 mM EDTA acid; pH 7.8) and then quantitated by a measurement of the optical density at 260 nm. The final preparation was stored at −20 °C and used as templates for polymerase chain reaction (PCR). Allele discrimination of the three studied SNPs was assessed with the ABI StepOne™ Real-Time PCR System (Applied Biosystems, Foster City, CA, USA) and analyzed using SDS version 3.0 software (Applied Biosystems, Foster City, CA, USA), with the TaqMan assay (Applied Biosystems, Foster City, CA, USA) [32].

### 2.4. Statistical Analysis

The demographic data and other clinically relevant data of continuous variables are presented as medians (interquartile range (IQR, 25th–75th percentile)) after Kolmogorov-Smirnov test for Normal distribution, whereas categorical variables are presented as numbers and percentages. Differences were compared between groups using the Kruskal Wallis test (for continuous variables) or chi-square test (for categorical variables) when appropriate. We used thef Bonferroni method as the post-hoc test for multiple comparisons after Kruskal Wallis test or chi-square test. The Hardy-Weinberg equation was used to calculate the expected numbers and then compared with the actual numbers of each phenotype. A chi-square test was performed to determine the Hardy-Weinberg equilibrium. The associations were examined between tested SNPs and overall ovarian stimulation outcomes under the different genetic models: genotypic model (AA versus Aa versus aa), dominant model (AA + Aa versus aa), and recessive model (AA versus Aa + aa). A more accurate estimate of the different effects of single-nucleotide polymorphisms on IVF outcomes was obtained by using multiple logistic regression models and adjusting for other covariates in IVF outcomes. All data were analyzed using the IBM SPSS Statistics for Windows, Version 22.0 (IBM Corp., Armonk, NY, USA). *p*-values < 0.05 were considered statistically significant.

## 3. Results

Allele frequencies for the AMH 146 T > G polymorphism were 59.6% for the T allele and 40.4% for the G allele. Genotype distribution was 35.0% homozygous T, 49.3% heterozygous and 15.7% homozygous G (Table 1). For the AMHRII −482A > G polymorphism, allele frequencies were 41% for the A allele and 59% for the G allele. The genotype distribution was 70.4% homozygous A, 27.6% heterozygous and 2.0% homozygous G (Table 2). Allele frequencies for the AMHRII IVS1 + 149 T > A polymorphism were 59.6% for the T allele and 40.4% for the A allele. The genotype distribution was 66.9% homozygous T, 30.1% heterozygous and 3.0% homozygous A (Table 3). The genotype distributions of these polymorphisms were consistent with the Hardy–Weinberg equilibrium, χ^2^ = 0.328, *p* = 0.849 for AMH 146 T > G; χ^2^ = 0.763, *p* = 0.683 for AMHRII −482 A > G; and χ^2^ = 0.032, *p* = 0.984 for AMHRII IVS1 +149 T > A.

A total of 635 women going through their first IVF treatment cycles were included in the study. Of the 635 patients, 357 (56.2%) were positive, and 278 (43.8%) had negative clinical pregnancy outcomes. There were no statistically significant differences in the AMH and AMHRII genotype frequencies concerning age, duration of infertility, basal AMH, total gonadotropin dose, number of oocytes retrieved, number of fertilized oocytes, number of good quality embryos, and number of transferred embryos (Table 1, Table 2 and Table 3). Except for one parameter, the AMH polymorphism, women with GG genotype presented higher basal FSH compared with TT genotype (7.01 versus 7.00 versus 6.10, *p* = 0.011; post hoc test: GG > TT, *p* = 0.003) (Table 1). The clinical pregnancy rate in the AMH 146 T > G T/T group was significantly higher than that in the G/G group (55.0% versus 51.8% versus 40.0%, *p* = 0.043; post hoc test: TT > GG, *p* = 0.013) (Table 1). However, no significant difference was shown between AMHRII polymorphisms and the clinical pregnancy outcomes of IVF (Table 2 and Table 3).

The genotype distribution and allele frequency of the AMH 146 T > G polymorphism between negative and positive groups are summarized in Table 4. Women with genotype GG were half as likely to have a clinical pregnancy compared with women with genotypes TT and TT/TG. However, no significant difference was found between genotypes TT and TG in pregnancy. A strong significant association was found between GG genotype and IVF outcome (OR = 0.55, 95% CI: 0.34–0.88, *p* = 0.014), which suggested that the AMH 146 T > G GG genotype decreased the chances of clinical pregnancy after IVF. No significant difference was found between both groups for the AMHRII −482 A > G and AMHRII IVS1 +149 T > A polymorphic genotypes (shown in Table 5 and Table 6). This means that the AMHRII −482 A > G and AMHRII IVS1 +149 T > A polymorphisms are not associated with pregnancy outcomes of IVF.

Univariate and multivariate logistic regression analysis and effects of variables to predict clinical pregnancy in IVF cycles are shown in Table 7. In the present study, the overall presentations are 2% and 3% for both AMHRII SNPs, which may not present statistical significance with conventional statistical testing. Consequently, these two SNPs were not included in the following analysis. Univariate logistic regression analysis showed that the AMH 146 T > G GG genotype (*p* = 0.014) was a factor that significantly predicted clinical pregnancy. As expected, women’s age (*p* = 0.001), number of oocytes retrieved (*p* < 0.001), and number of oocytes in metaphase II retrieved (*p* < 0.001) were also factors that significantly predicted clinical pregnancy. With multivariate analysis, the AMH 146 T > G GG genotype remains as a significant independent factor to predict clinical pregnancy (*p* = 0.014).

## 4. Discussion

This study explored polymorphisms of AMH and AMHRII to obtain new insights about their association with IVF outcomes. We found that infertile women carrying the genotype (GG) at AMH 146 T > G had lower IVF pregnancy rates. The distribution frequency of AMH GG genotype was lower in positive pregnancy patients than in negative pregnancy patients. To the best of our knowledge, our study is the first to report for an Asian population whether AMH polymorphism can affect pregnancy rates in women receiving their first IVF cycles. However, no significant difference was found between positive and negative pregnancy women undergoing IVF treatment for AMHRII −482 A > G and AMHRII IVS1 +149 T > A polymorphism.

It is interesting to noted that the distribution of AMH 146 T > G in the present study in Taiwan (TT 35%, TG 49.3%, GG 15.7%) is quite different from those reported in Greece (TT 64.2%, TG 33.1%, GG 2.6%) [21] and in the UK (TT 63%, TG 33%, GG 4%) [28] for women undergoing IVF treatment. By contrast, the distribution of AMH 146 T > G in the present study was more similar to that reported in Japanese women (TT 37.5%, TG 50.0%, GG 12.5%) [29]. The G allele in AMH 146 T > G SNP was much more present in Taiwan population than those in Greece and UK. This difference may, at least partially, explain the different finding between our results and that reported by Cerra et al. in 2016 [28] regarding the relevance of AMH 146 T > G to the pregnancy outcome in IVF cycles. The AMHRII −482 A > G SNP distribution is very similar among the three groups (AA 70.4%, AG 27.6%, GG 2.0% in Taiwan, AA 69.6%, AG 29.1%, GG 1.4% in Greece and AA 69%, AG 28%, GG 3% in UK). Nonetheless, the AMHRII SNP genotype frequency is a little different from those in Japanese women (AA 50.9%, AG 40.4%, GG 8.7%) [29]. In the report in Japan, AMHRII −482 A > G were associated with follicular development and poor responders in IVF cycles. The more common G allele of AMHRII −482 A > G in Japanese women may also account for the association between the AMHRII SNP and follicular development in Japanese report.

Our results revealed similar findings with those reported in Greece [21]: the G/G genotypes of the AMH polymorphism demonstrated significantly higher basal FSH levels (*p* = 0.011) (Table 1). A possible explanation for the higher basal FSH levels in the AMH 146 T > G G/G genotype may be the impaired function of AMH molecule produced by such genotypes. Such impaired AMH might accelerate recruitment of primordial follicles leading to an exhaustion of follicles from the primordial follicle pool. High basal FSH levels display the decline of ovarian reserve and are associated with a reduced fecundity [18,33]. The impaired function of AMH translated from G/G genotype might be connected with the phenotype of low AMH levels, although the serum AMH levels detected by immunoassay are not affected. Our previous study suggested that the effect of low serum AMH levels on pregnancy outcome in IVF cycles might be associated with follicular FSH levels [12]. High follicular FSH levels might be correlated with aberrant meiosis and high aneuploid rates of oocytes in IVF cycles [34,35]. The AMH 146 T > G G/G genotype is also associated with non-significant fewer mature oocytes (*p* = 0.094, Table 1), which indicate that the final oocyte meiosis might be affected by such dysfunctional AMH. It has been verified that oocyte aneuploidy is the main cause for implantation failure or decreased fertility in older women [36]. Furthermore, infertile patients with decreased ovarian reserve exhibit higher percentages of aneuploid blastocysts [37,38,39]. Taking together, the G/G genotype of AMH 146 T > G is associated with the phenotype of low AMH in IVF cycles (high FSH levels and low pregnancy rates).

Concerning the AMHRII polymorphisms, most studies about IVF focused on ovarian reserve or ovarian response to draw some conflicting conclusions [25,26,27,28]. In our prospective study of 635 women, genotyping of the AMHRII −482 A > G, and AMHRII IVS1 +149 T > A polymorphisms does not provide additional useful information as a predictor of ovarian response. The AMHRII −482 A > G G/G genotype is associated with high LH levels, non-significantly high FSH levels, and non-significantly low pregnancy rates. The polymorphism G/G genotype features only 2% of the test sample. It is difficult to draw firm conclusion for non-significant difference for <5% SNP polymorphism in the population by conventional statistical methods. By contrast, the AMHRII −482 A > G G/G genotype is more common in Japanese women compared to that in Taiwanese women and the G/G genotype is associated with poor responders in Japanese women. The AMHRII-482 SNP was worthy of further investigation [29]. In addition, the pattern of FSH/LH and pregnancy outcome for AMHRII −482 A > G G/G phenotype is similar with those for AMH 146 T > G G/G genotype. We could not exclude the possibility that AMHRII −482 A > G G/G genotype is associated with dysfunction of AMH/AMHRII signaling in ovarian granulosa cells and reduced pregnancy rates in IVF cycles. Further biological mechanism investigation is needed to prove the concept. 

The limitations of the present study were the lack the embryo-related genotype analysis, the recruitment of Han Chinese people only and the lack of access to the advanced statistical testing for polymorphism frequency less than 5%. Due to the effect of ethnicity on SNP polymorphism frequency, the results of the present study may be not applicable for the population of western countries. The frequency of polymorphism genotype for AMHRII −482 A > G and IVS1 +149 T > A is only 2.0% and 3.0%, respectively, which may not present statistical significance with conventional statistical testing. To further delineate the effect of AMHRII SNP, we will have to recruit more subjects or use more powerful testing, such as generalized regression with machine-learning based analytics.

## 5. Conclusions

In conclusion, the results demonstrated that the association of the AMH 146 T > G (rs10407022) polymorphism with IVF outcome and the GG genotype might lower the chance of getting pregnant than compared with the others. Further research with a larger sample size or biological mechanism needs to be undertaken to verify our results.

## Figures and Tables

**Table 1:** Baseline characteristics and in vitro fertilization (IVF) treatment cycle outcomes of patients with different AMH 146 T > G (rs10407022) SNP.

Total *n* = 635	TT	TG	GG	*p*-Value
	*n* = 222 (35.0%)	*n* = 313 (49.3%)	*n* = 100 (15.7%)	
Age (years)	35 (32–38)	35 (33–38)	35 (31–38)	0.473
Duration of infertility (years)	2.5 (2–5)	2.5 (1.5–4)	3 (2–4)	0.646
Basal FSH (mIU/mL)	6.10 (4.47–8.36)	7.00 (5.30–8.70)	7.01 (4.75–8.42)	0.011
Basal LH (mIU/mL)	4.21 (3–6.65)	4.90 (3.22–7.40)	4.67 (3.00–7.02)	0.110
Basal AMH (mIU/mL)	3.18 (1.17–5.80)	2.95 (1.39–5.60)	2.54 (1.24–5.39)	0.681
Basal estradiol (pg/mL)	26 (18–48)	27 (19–47)	27 (21–47.5)	0.670
Estradiol on HCG day (pg/mL)	2095 (1173–3767.5)	1811 (1005–3063.5)	2106.5 (1020–3397.5)	0.215
Progesterone at HCG day (ng/mL)	1.03 (0.64–1.4)	0.98 (0.61–1.34)	1.04 (0.76–1.55)	0.199
Duration of stimulation (days)	10 (9–11)	10 (9–11)	10 (9–11)	0.286
Total gonadotropins dose (IU)	2775 (2550–3300)	2775 (2550–3450)	2775 (2550–3075)	0.364
Number of oocytes retrieved	11 (6–18)	10 (5.5–16)	10 (5.5–16.5)	0.468
Under–response (<4 oocytes)	31/222 (14%)	39/313 (12.5%)	16/100 (16%)	0.658
Over–response (>20 oocytes)	41/222 (18.5%)	46/313 (14.7%)	15/100 (15%)	0.487
Number of mature oocytes	9 (4–15)	8 (4–12)	8 (4–12.5)	0.094
Number of day 3 embryos	7 (3–12)	6 (3–10)	6 (3–11)	0.127
Fertilization rate (%)	87.50 (72.73–100)	90.00 (75–100)	90.91 (75–100)	0.352
Day 3 good embryo rate (%)	56.35 (40–72.48)	57.14 (38.46–72.22)	60.00 (45.45–75.00)	0.506
Day 5 good embryo rate (%)	47.06 (31.25–58.82)	44.28 (25–57.14)	40.00 (33.33–55.56)	0.586
Endometrial thickness(mm)	12 (10–13)	12 (10–13)	12 (10–14)	0.420
Number of transferred embryos	2 (2–3)	2 (2–3)	2 (2–3)	0.747
Clinical pregnancy	122/222 (55.0%)	162/313 (51.8%)	40/100 (40.0%)	0.043

*p*-value by Kruskal Wallis test, chi-square test or Fisher’s exact test when appropriated, and followed with Bonferroni procedure for multiple comparisons. IVF: in vitro fertilization; AMH: anti-Müllerian hormone; SNP: single-nucleotide polymorphism.

**Table 2:** Baseline characteristics and IVF treatment cycle outcomes of patients with different AMHRII −482 A > G (rs2002555) SNP.

Total *n* = 635	AA	AG	GG	*p*-Value
	*n* = 447 (70.4%)	*n* = 175 (27.6%)	*n* = 13 (2.0%)	
Age (years)	35 (32–38)	35 (32–38)	33 (30–36)	0.470
Duration of infertility (years)	2.5 (2–4)	3.0 (2–5)	2.5 (1.5–3)	0.549
Basal FSH (mIU/mL)	6.70 (5.00–8.65)	6.80 (4.50–8.36)	6.87 (6.30–8.70)	0.594
Basal LH (mIU/mL)	4.69 (3.20–7.23)	4.10 (2.89–6.40)	6.80 (5.30–7.40)	0.006
Basal AMH (mIU/mL)	3.06 (1.26–5.70)	2.84 (1.12–5.56)	2.30 (1.30–6.60)	0.609
Basal estradiol (pg/mL)	28 (20–48)	25 (17–45)	27 (18–43)	0.283
Estradiol on HCG day (pg/mL)	1937.5 (1098–3465)	2049 (1031–3113)	1495.5 (966–2422.5)	0.540
Progesterone at HCG day (ng/mL)	1.00 (0.66–1.35)	1.08 (0.62–1.48)	0.81 (0.57–1.08)	0.351
Duration of stimulation (days)	10 (9–11)	10 (9–11)	10 (9–10)	0.269
Total gonadotropins dose (IU)	2775 (2550–3300)	2775 (2550–3300)	2775 (2550–3975)	0.714
Number of oocytes retrieved	10 (6–17)	11 (6–17)	9 (6–11)	0.572
Under–response (<4 oocytes)	57/447 (12.8%)	28/175 (16%)	1/13 (7.7%)	0.453
Over–response (>20 oocytes)	77/447 (17.2%)	24/175 (13.7)	1/13 (7.7%)	0.409
Number of mature oocytes	8 (4–13)	8 (3–14)	7 (4–10)	0.507
Number of day 3 embryo	7 (3–11)	6 (3–11)	6 (3–8)	0.649
Fertilization rate (%)	88.89 (75–100)	90.00 (71.43–100)	90.00 (78.95–100)	0.865
Day 3 good embryo rate (%)	57.14 (40–75)	57.14 (40.63–72)	61.12 (35.42–79.17)	0.928
Day 5 good embryo rate (%)	44.44 (28.57–57.14)	42.86 (25.00–55.56)	45.45 (33.33–66.67)	0.585
Endometrial thickness(mm)	12 (10–13)	11 (10–13)	12 (11–14)	0.481
Number of transferred embryos	2 (2–3)	2 (2–3)	2 (2–3)	0.929
Clinical pregnancy	227/447 (50.8%)	93/175 (53.1%)	4/13 (30.8%)	0.292

*p*-value by Kruskal Wallis test, chi-square test or Fisher’s exact test when appropriated. FSH: follicle-stimulating hormone; LH: luteinizing hormone; HCG: human chorionic gonadotropin; IU: International unit.

**Table 3:** Baseline characteristics and IVF treatment cycle outcomes of patients with different AMHRII IVS1 +149 T > A (rs2272002) SNP.

Total *n* = 635	TT	TA	AA	*p*-Value
	*n* = 430 (67.7%)	*n* = 186 (29.3%)	*n* =19 (3.0%)	
Age (years)	35 (32–38)	35 (32–38)	34 (32–38)	0.953
Duration of infertility (years)	3 (2–5)	2 (1–4)	3 (2–4)	0.131
Basal FSH (mIU/mL)	6.80 (4.84–8.50)	6.63 (4.97–8.80)	6.50 (5.30–8.33)	0.935
Basal LH (mIU/mL)	4.51 (3.10–6.80)	4.67 (3.10–7.40)	5.07 (3.96–9.34)	0.285
Basal AMH (mIU/mL)	2.89 (1.26–5.55)	3.2 (1.13–5.82)	4.67 (2.50–6.80)	0.420
Basal estradiol (pg/mL)	26 (19–47)	29 (18–48)	30 (22–48)	0.440
Estradiol on HCG day (pg/mL)	1932.5 (1109.5–3191.5)	1935 (967–3451)	3712 (938–4217)	0.483
Progesterone at HCG day (ng/mL)	0.98 (0.63–1.36)	1.11 (0.67–1.40)	0.69 (0.45–1.61)	0.364
Duration of stimulation (days)	10 (9–11)	10 (9–10)	10 (9–11)	0.242
Total gonadotropins dose (IU)	2775 (2550–3300)	2775 (2550–3300)	2775 (2550–3075)	0.630
Number of oocytes retrieved	10 (6–16)	11 (5–17)	12 (4–19)	0.946
Under-response (<4 oocytes)	55/430 (12.8%)	27/186 (14.5%)	4/19 (21.1%)	0.534
Over-response (>20 oocytes)	68/430 (15.9%)	30/186 (16.1%)	4/19 (21.1%)	0.833
Number of mature oocytes	8 (4–13)	8 (4–13)	9 (3–15)	0.968
Number of day 3 embryo	6 (3–11)	7 (3–12)	7 (3–14)	0.911
Fertilization rate (%)	88.89 (71.43–100)	90.91 (77.78–100)	93.33 (77.78–100)	0.128
Day 3 good embryo rate (%)	57.14 (40.00–75.00)	57.14 (40.00–71.43)	50.00 (33.33–66.67)	0.684
Day 5 good embryo rate (%)	45.23 (30.77–57.14)	43.62 (25.00–55.56)	40.00 (33.33–60.87)	0.513
Endometrial thickness(mm)	12 (10–13)	12 (10–13)	11 (10–13)	0.754
Number of transferred embryos	2 (2–3)	2 (2–3)	2 (2–3)	0.996
Clinical pregnancy	215/430 (50.0%)	98/186 (52.7%)	11/19 (57.9%)	0.689

*p*-value by Kruskal Wallis test, chi-square test or Fisher’s exact test when appropriated.

**Table 4:** Frequencies of AMH 146T > G (rs10407022) polymorphism among women with different IVF outcome.

		Clinical Pregnancy				Univariate Analysis		
		Negative		Positive				
		*n*	%	*n*	%	Odds Ratio	95% Confidence Interval (CI)	*p*-Value ^a^
Codominant	TT	100	32.2	122	37.7	Reference		
	TG	151	48.6	162	50.0	0.879	0.623–1.242	0.465
	GG	60	19.3	40	12.3	0.546	0.338–0.883	0.014
Dominant	TT/TG	251	80.7	284	87.7	Reference		
	GG	60	19.3	40	12.3	0.589	0.382–0.910	0.017
Recessive	TT	100	32.2	122	37.7	Reference		
	TG/GG	211	67.8	202	62.3	0.785	0.566–1.089	0.147
Allele	T	351	56.4	406	62.7	Reference		
	G	271	43.6	242	37.3	0.772	0.617–0.966	0.024

^a^*p*-value by logistic regression analysis.

**Table 5:** Frequencies of AMHRII −482 A > G (rs2002555) polymorphism among women with different IVF outcome.

		Clinical Pregnancy				Univariate Analysis		
		Negative		Positive				
		*n*	%	*n*	%	Odds Ratio	95% CI	*p*-Value ^a^
Codominant	AA	220	70.7	227	70.1	Reference		
	AG	82	26.4	93	28.7	1.099	0.775–1.560	0.597
	GG	9	2.9	4	1.2	0.431	0.131–1.419	0.166
Dominant	AA/AG	302	97.1	320	98.8	Reference		
	GG	9	2.9	4	1.2	0.419	0.128–1.376	0.152
Recessive	AA	220	70.7	227	70.1	Reference		
	AG/GG	91	29.3	97	29.9	1.033	0.735–1.453	0.852
Allele	A	522	83.9	547	84.4	Reference		
	G	100	16.1	101	15.6	0.964	0.713–1.303	0.811

^a^*p*-value by logistic regression analysis.

**Table 6:** Frequencies of AMHRII IVS1 +149 T > A (rs2272002) polymorphism among women with different IVF outcome.

		Clinical Pregnancy				Univariate Analysis		
		Negative		Positive				
		*n*	%	*n*	%	Odds Ratio	95% CI	*p*-Value ^a^
Codominant	TT	215	69.1	215	66.4	Reference		
	TA	88	28.3	98	30.2	1.114	0.789–1.571	0.540
	AA	8	2.6	11	3.4	1.375	0.542–3.485	0.502
Dominant	TT/TA	303	97.4	313	96.6	Reference		
	AA	8	2.6	11	3.4	1.331	0.528–3.354	0.544
Recessive	TT	215	69.1	215	66.4	Reference		
	TA/AA	96	30.9	109	33.6	1.135	0.814–1.584	0.455
Allele	T	518	83.3	528	81.5	Reference		
	A	104	16.7	120	18.5	1.132	0.848–1.512	0.401

^a^*p*-value by logistic regression analysis.

**Table 7:** Univariate and multivariate logistic regression analysis of different variables to predict clinical pregnancy after IVF cycles.

Predictor	Univariate Analysis			Multivariate Analysis		
	Odds Ratio	95% CI	*p*-Value	Odds Ratio	95% CI	*p*-Value
Age	0.934	0.899 0.971	0.001	0.955	0.916–0.996	0.031
Basal AMH (mIU/mL)	1.108	1.056–1.163	<0.001	1.065	0.995–1.140	0.069
Number of oocytes retrieved	1.036	1.016–1.055	<0.001	1.010	0.984–1.037	0.450
Number of mature oocytes	1.048	1.024–1.071	<0.001			
Number of embryo transfer	1.106	0.921–1.329	0.278			
AMH 146 T > G (TT versus GG)	1.830	1.133–2.956	0.014	1.852	1.134–3.022	0.014
AMH 146 T > G (TG versus GG)	1.609	1.019–2.543	0.040	1.681	1.053–2.685	0.030
